# Synchronous cemento-ossifying fibromas: a systematic review

**DOI:** 10.4317/medoral.26501

**Published:** 2024-08-01

**Authors:** Bruna Luísa Neumann, Eduarda Martins Mendes, Bruna Barcelos Só, Felipe Martins Silveira, Vivian Petersen Wagner, Pablo Agustin Vargas, Jean Nunes Dos Santos, Felipe Paiva Fonseca, Ronell Eduardo Bologna-Molina, Adalberto Mosqueda-Taylor, Marco Antonio Trevizani Martins, Manoela Domingues Martins, Lauren Frenzel Schuch

**Affiliations:** 1 Department of Oral Pathology, School of Dentistry, Federal University of Rio Grande do Sul, Porto Alegre, RS, Brazil; 2 Department of Diagnosis in Pathology and Oral Medicine, School of Dentistry, Universidad de la República, Montevideo, Uruguay; 3 Department of Dentistry, School of Dentistry, Universidade de São Paulo (FOUSP), São Paulo, Brazil; 4 Department of Oral Diagnosis, Piracicaba Dental School, Campinas University, Piracicaba, SP, Brazil; 5 Department of Oral Pathology, School of Dentistry, Federal University of Bahia, Salvador, BA, Brazil; 6 Department of Oral Surgery and Pathology, School of Dentistry, Universidade Federal de Minas Gerais, Belo Horizonte, Brazil; 7 Health Care Department, Universidad Autónoma Metropolitana Xochimilco, Ciudad de México; 8 Department of Oral Medicine, Hospital de Clínicas de Porto Alegre (HCPA/UFRGS), Porto Alegre, Rio Grande do Sul, Brazil

## Abstract

**Background:**

This systematic review aimed to incorporate published data regarding synchronous cemento-ossifying fibromas (COF), with an analysis of their demographic and clinicopathological characteristics.

**Material and Methods:**

Case reports and case series of synchronous COF were searched in PubMed, Web of Science, Scopus, EMBASE, and LILACS according to the PRISMA (2020) statement. Also, a manual search was carried out and the grey literature was assessed. A descriptive statistical analysis was performed.

**Results:**

Nineteen studies comprising 20 cases of synchronous COF were included. The mean age at diagnosis was 35 years (±13.8), with a predominance of female patients (*n*=12/60%). In 13 cases (65%) the mandible and the maxilla were affected simultaneously. In two cases (10%) first-degree relatives (parents or siblings) had been previously diagnosed with COF. The diagnostic hypotheses were reported in 8 cases (40%), with florid cemento-osseous dysplasia, ameloblastic fibroodontoma, calcifying cystic odontogenic tumor, osteoma and cementoblastoma being cited in the differential diagnosis. Among the cases with details about management (*n*=17), eleven were treated by surgical enucleation and/or excision (64.7%). Follow-up was provided for 10 cases (50%), with a mean period of 44.7±62.19 months. Recurrence occurred in three of informed cases.

**Conclusions:**

Synchronous manifestation of COF is rare. Female patients around the 3rd decade of life are more commonly affected. Bilateral involvement of the mandible and maxilla is the most common clinical presentation.

** Key words:**Ossifying fibroma, cemento-ossifying fibroma, synchronous, oral pathology.

## Introduction

Cemento-ossifying fibroma (COF) is a well-recognized benign neoplasm that affects the jaw bones. Since it is composed of fibrocellular tissue and varying amounts of mineralized material resembling bone and/or cementum, it has been classified as a benign fibro-osseous lesion (1). Based on the 2022 WHO classification, it is currently described as a benign mesenchymal odontogenic tumor and as a separate entity from the non-odontogenic juvenile trabecular and psammomatoid types (2). Some other names previously used for this lesion include central ossifying fibroma, cementifying fibroma, and periodontoma (3). The pathogenesis of COF in a subset of tumors has been linked to inactivating mutations in the tumor suppressor gene CDC73 (HRPT2), most commonly in cases of hyperparathyroidism-jaw tumor (HPT-JT) syndrome. COF can also be associated with gnathodiaphyseal dysplasia, which is defined by GDD1 gene mutations (2).

COF is considered to be a rare lesion with an incidence peak around the third and fourth decades of life and with female predilection. COF occurs exclusively in the tooth bearing areas of the mandible and maxilla, with the mandible, especially the premolar and molar area, being more commonly affected than the maxilla (3,4). Clinically, COF usually present as a painless expansion of the buccal and lingual plates of the affected bone. Most COF is slow-growing solitary intraosseous lesions, being frequently discovered as incidental findings. Radiographically, early lesions are typically radiolucent, with the tumors becoming progressively more radiopaque over time. Treatment can be conservative enucleation, with no recurrence in most cases, while resection has been chosen in more extensive lesions (3,5).

COF may rarely involve the jaws bilaterally or multiple quadrants and are considered synchronous when two or more lesions occur simultaneously within an interval of 6 months or less (6). Synchronous COF is uncommon, with scarce examples scattered across the literature. The objective of the present study was to integrate in a systematic review published data about synchronous COF, considering their demographic, clinical, imaging, and histopathological characteristics, in addition to their treatment, follow-up and frequency of recurrence.

## Material and Methods

- Information sources and search strategies

Two examiners (E.M.M. and B.L.N.) independently conducted electronic searches on the following databases: PubMed, Web of Science, Scopus, EMBASE, and LILACS, considering eligibility criteria (English language, with no restrictions of publication year) in July 2022 and updated in April 2023. The search strategy was based on the Population, Exposure, Comparison Outcomes, and Study Design (PECOS) principle, combining Medical Subject Heading (MeSH) terms and text words with Boolean operators "AND" and "OR" and adapting to the syntax rules of each database (Supplement 1). Additional manual searches were conducted from the reference lists of the included studies. EndNote X9 software (Thomson Reuters, Philadelphia, PA) was used to manage and organize the references, including removal of duplicates.

- Eligibility criteria

All case reports and case series classified as synchronous COF and within the PECOS principle (7) were included. The PECOS principle was applied to the current case report review, as follows: (P) Population, patients; (E) Exposure, synchronous COF; (O) Outcome, clinicopathological data regarding COF; and (S) Study design, case reports and case series.

Articles describing case reports or case series of synchronous COF (e.g., simultaneous lesions or lesions separated by an interval of up to six months (6) located at intraoral sites, on the maxilla and mandible with clinical, radiological, and histopathological information (clear description of the morphological findings and/or histopathological photomicrographs confirming the diagnosis) were included. Articles that did not provide a histopathological photomicrograph and/or a clear description of the morphological findings in order to establish the diagnosis were excluded. Also excluded were letters to the editor, review articles, comments, congress abstracts, opinions, and laboratory studies, publications in languages other than English, and articles whose full text was not available.

- Study selection and data extraction

Two independent authors (E.M.M. and B.L.N.) carried out the study selection process by searching electronic databases and importing them into the reference manager. The duplicates were then removed, and the titles and abstracts were analyzed. The full texts of the selected studies were then accessed based on the eligibility criteria. In the event of a disagreement over study inclusion, a third reviewer was consulted (L.F.S.). Next, all cases were critically reviewed. If they did not provide high-quality radiographic and histopathological images, if the images did not look convincingly like the proposed diagnosis, or if images of each of the synchronous lesions were not provided, the article was removed from consideration. When there were questions about the diagnoses, the authors of the articles were contacted via email.

Two authors (E.M.M. and B.L.N.) worked independently on data extraction. The following information was gathered from the included articles: author's name, publication date, country, patient sex, age and clinical condition, anatomical location of the lesions, bi- or multifocal occurrence, affected side(s), affected jaw bones (mandible, maxilla or both), radiographic features, histopathological features, treatment, follow-up, and recurrence. 

- Quality assessment of the included studies

Critical appraisal of the included articles was carried out by means of the Joanna Briggs Institute - University of Adelaide tool for case reports and case series (8,9). Quality was assessed by two independent authors (B.L.N. and E.M.M.) in order to determine the risk of bias of each study. Whenever differences occurred, a third author was consulted (L.F.S.). Articles were evaluated according to the following parameters: clear description of patient’s characteristics, medical history and current clinical condition, clear description of the propaedeutic data, treatment, post-intervention clinical condition, adverse events, and lessons provided by the case report.

- Protocol and registration

This systematic review was conducted in accordance with the Preferred Reporting Items for Systematic Review and Meta-Analysis (PRISMA) (10) recommendations and was registered in the Prospective Register of Systematic Reviews (PROSPERO; Center for Reviews and Dissemination, University of York) under registration number CRD42021238295.

## Results

- Study selection

**Figure 1:**
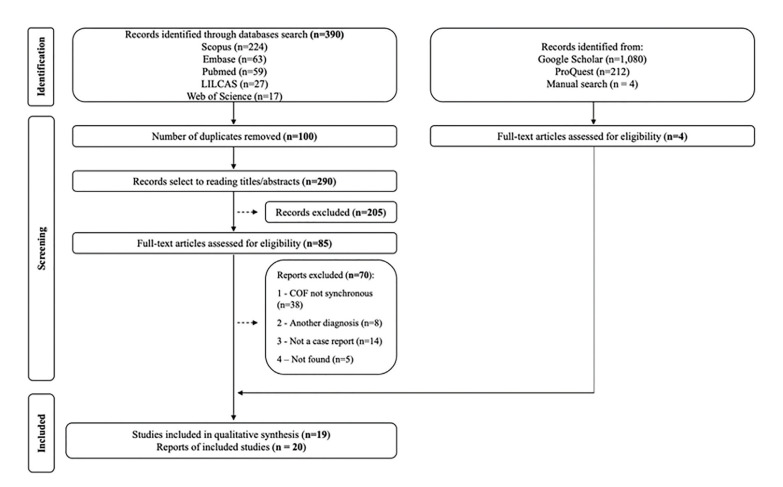
PRISMA flow diagram for a systematic search and study selection.

Fig. 1 presents the flowchart of the search procedure. A total of 390 potentially pertinent records were retrieved from the electronic databases. After duplicates were removed, 290 records were reviewed based on their titles and abstracts. Of these, 205 were excluded for not fitting the eligibility criteria. Eighty-five articles were assessed for full text reading and 70 papers were excluded for different reasons (Supplement 2). A manual search of the listed papers' bibliographies yielded four additional studies. Finally, this systematic review comprised a total of 19 articles (11-29) reporting a total of 20 occurrences of Synchronous COF.

- General characteristics of the included studies

Cases were published in nine different countries, most of them in India (*n*=4/20%), followed by Turkey and the US with three cases (15%) each. The studies were published between 1968 and 2015. Supplement 3 and Table 1 provide clinical information from the included studies.

Mean age at diagnosis was 35 years (±13.87), and females (n = 12; 60%) were more affected than males (n = 8; 40%) with a male-to-female ratio of 1:1.5.

Of the 20 cases included in the analysis, 4 did not provide detailed information regarding patients' previous medical history, while 3 cases just reported the absence of noteworthy health conditions. In the remaining 13 cases, a more specific approach was undertaken, delineating the types of tests conducted and the resultant findings. It is imperative to underscore that all cases ruled out the presence of syndromes. Among the analyzed cases, three exhibited abnormalities: elevated alkaline phosphatase; hypertension, hypokalemia, and proteinuria, as well as primary hyperaldosteronism attribuTable to an adrenal gland tumor; and low hemoglobin levels.

Regarding clinical data, 14 cases (70%), were asymptomatic, and 3 (15%) reported painful symptoms. Furthermore, 12 cases (60%) reported facial asymmetry such as cortical expansion. The clinical appearance of the lesions was reported in 16 cases (80%) and described as a hardened swelling in most cases. The mucosa was described as normal in 12 cases (60%) and eight cases (40%) did not report this information.

Regarding the radiographic findings, 17 cases (85%) described lesions with well-defined margins. Twelve cases (60%) described them as mixed lesions (radiolucent with radiopaque areas), six (30%) as radiolucent lesions, and two (10%) as radiopaque lesions. Two cases (10%) reported that the lesions contained calcifications with the appearance of snowflakes. About adjacent structures, nine cases (45%) reported root resorption, six (30.0%) revealed cortical expansion, and seven (35%) reported extension to the sinuses.

In general, COF mostly affected both jaws (13 cases; 65%), only the mandible in five cases (25%), and only the maxilla in two cases (10%). Overall, seventeen cases (85%) had two synchronous lesions and 3 cases (15%) had more than two lesions, two of them with 3 lesions and one with 5 lesions (in four jaw quadrants).

Regarding patients' medical history, one patient had primary hyperaldosteronism attribuTable to an adrenal gland tumor. Two cases (10%) reported a family history of first-degree relatives (parents or siblings) previously diagnosed with COF. Serum tests were performed in 3 cases and all were normal.

The diagnostic hypotheses were reported in 8 cases (40%). In four of them, a provisional diagnosis of COF was made, whereas ossifying fibroma and florid cemento-osseous dysplasia were reported in one case each. One case had a suspicious of COF or residual cyst and the other of calcifying odontogenic cyst or ameloblastic fibro-odontoma.

Among the cases that reported treatment (*n*=17), eleven were treated by surgical enucleation and/or excision (64.7%), four adopted resection as the treatment of choice (23.5%), one was treated only with symptomatic therapy (5.8%) since the patient refused surgical treatment because of complete pain relief by therapy, and in one case treatment was not started due to the patient's choice (5.8%).

Three patients experienced recurrences. All cases occurred in women between 19 to 31 years old over a period ranging of 6 months to 2 years. One of the cases involved a lesion of considerable dimensions, weighing approximately 1.5 kg (18). The other two cases reported elevated alkaline phosphatase levels, of 278 IU/L (28) and 218 IU/L (29).

Follow- up was reported in 10 cases with a mean of 44.7 (±62.19) months.

- Quality assessment

All articles gave clear descriptions of the patient's demographic characteristics, and most papers also described the patient's current clinical presentation and the diagnostic procedures or methods used. In contrast, most of the included publications poorly characterized the patients' histories and offered no actionable advice. Supplement 4 describes the evaluation of risk of bias in more detail.

## Discussion

COF is a true benign neoplasm, well defined and composed of fibrocellular tissue and of mineralized material of variable appearance (3,30). It mainly affects the mandible and, if not treated, can show continuous growth (2). Synchronic lesions (i.e., two or more lesions occurring simultaneously) are rare and, to be classified as such, they must be located at different sites and be separated by a maximum interval of six months (6), and these have to exclude hyperparathyroidism-jaw tumor (HPT-JT) syndrome and gnathodiaphyseal dysplasia. Twenty cases of synchronous COF were detected in the present review, showing clinical and behavior features of this lesion.

According to the literature, COF occurs more frequently in young adults, between the third and fourth decades of life, with female predilection (30,31). In the present study, the occurrence of synchronous COF was higher among female patients with a mean age of 35 years (±13.87). Clinically, isolated COF is most often found in the posterior mandible (30). However, in this review, the synchronous lesions occurred more commonly in both jaws.

Regarding the medical history of the patients, one case described hyperaldosteronism and 3 cases provided information about normal serum tests, excluding the presence of HPT-JT syndrome. The occurrence of COF may be associated with syndrome the HPT-JT (12). Unlike the brown tumor of hyperparathyroidism, COF lesions do not regress after surgical treatment of the parathyroid (32). However, it is crucial to check the levels of serum calcium, phosphorus, and parathyroid hormone in order to distinguish between synchronous COF and HPT-JT syndrome (13). The serum levels of alkaline phosphatase of the patient reported by Yih *et al*. (29) increased over the course of the patient's follow-up. It is interesting to note that the cited authors also discovered a COF in the left mandible and another in the maxilla of the patient's mother. Unfortunately, although the family history was strongly suggestive of HPT-JT, the authors did not investigate serum PTH levels, with a possible misinterpretation of their case. According to Ribeiro *et al*. (23), the analysis of serum PTH levels is the most important test to be applied to rule out a potential relation to HPT-JT, being essential for a correct diagnosis. Regarding the hyperaldosteronism of the case reported by Sakuma *et al*. (24), there is no evidence linking this condition to COF in the literature.

The discovery of these lesions can be incidental, as a radiographic finding during routine examination, or after patients seek evaluation in cases of cortical bone expansion. Chang *et al*. (33) reported that the most frequent clinical sign of COF was bone swelling or expansion of the buccal and/or lingual cortical plates. In the present review, cortical expansion was reported in six cases (30%). Depending on the level of mineralization, COF may exhibit various radiographic patterns. It first appears as a radiolucent lesion and, as the lesion develops, the calcified foci grow until the lesion becomes fully radiopaque. The primary distinguishing feature of COF is its eccentric growth pattern, which causes it to spread out in all directions and eventually create a noticeable spherical mass that can be distinguished from the surrounding tissues (1). According to Titinchi and Morkel (5), COF is radiopaque in about 50% of cases. However, Shirafkan *et al*. (1) reported that only 15% of COF cases present as solely radiolucent, while 35% present as radiolucent-radiopaque lesions. Most of the reports reviewed here described the lesions as well-defined and with a mixed pattern (radiolucent with radiopaque areas).

Histopathologically, COF is a well-defined lesion, usually encapsulated and composed of hypercellular fibroblastic stroma containing, at most, variable amounts of calcified structures resembling bone and/or cementum, and therefore it has been classified as a benign fibro-osseous lesion (3). In most of the cases reviewed here, the lesions were described as fibroblastic stroma with small calcifications. However, it is important to emphasize that the correlation of clinical, radiographic, and histopathological findings is essential for a correct diagnosis.

Our findings demonstrated that patients with synchronous COF were usually treated by enucleation surgery without a margin approach. Since this is a benign lesion with low recurrence rates (3), conservative surgical removal is usually the selected treatment option. Enucleation is one of the recommended treatments for small lesions, whereas curettage is recommended for lesions without a clear radiolucency surrounding them, and *en bloc* resection combined with bone regeneration is recommended for large lesions close to the inferior border of the mandible (1,3). In more advanced cases, these lesions can affect other structures of the face, such as the ethmoid bone and sinuses, requiring more invasive and mutilating treatment, such as maxillary or mandibular resection (5,17-19). It is also interesting to note that seven cases included in our sample showed sinus expansion, raising questions about the true origin of the lesion. Given that COF originates in the periodontal membrane, the literature contends that it is illogical for it to exist in anatomical locations unrelated to the periodontal membrane (14,34). It was believed that ectopic periodontal membranes exist and that pluripotent mesenchymal cells may develop in addition to the periodontal ligament to form calcified material resembling bone and cementum (14,34).

Follow-up was reported only in 50% of the cases. In three cases, patients refused treatment. Recurrence lesion was reported in 3 (23.1%) of 13 informed cases, and all of them affected female patients, between 19 to 31 years old, during a period ranging from 6 months to 2 years. Two of the recurrence cases occurred in patients with elevated alkaline phosphatase, despite normal serum levels of calcium and sodium. The literature indicates that individuals with recurrent lesions had the highest mean serum alkaline phosphatase level compared to those with established fibro-osseous lesions (35). The other cases occurred in a lesion with 1.5kg of weight (18), and recurrence may have occurred due to incomplete tumor removal. However, it is important to highlight that this recurrence among reported cases and the patients’ characteristics need to be interpreted with caution since these results might have been influenced by the small sample size. Also, reporting/publication bias needs to be taken into account since it is possible that cases that recurred are more commonly published than those that did not.

The current review has some limitations. Due to the rarity of these lesions, the sample size was necessarily limited. Moreover, some important variables were poorly reported in the studies, such as a medical description of the patients and little information about follow-up. In this respect, it is important to point out that case reports and case series follow reporting standards, such as CARE guidelines, in order to standardize data and preserve case specifics (36). To further understand the clinicopathologic, radiologic, prognostic, therapeutic, and recurrence aspects of synchronous COF, additional retrospective multicenter studies with sizable samples should be carried out.

## Conclusions

This review compiled 20 cases of synchronous COF. We can conclude that these lesions are rare, mostly affecting women in the 3rd decade of life, that they are localized in the maxilla and mandible, being asymptomatic and showing expansive growth. The treatment was surgical enucleation or excision, recurrence appear in four cases, and only in three patients’ serum tests were realized. Although this analysis clarified some of the key aspects of synchronous COF, its etiopathogenesis remains unexplained, supporting the need for additional research. When confronted with comparable difficult instances during clinical practice, the oral surgeon may use the guidelines provided for synchronous COF in the present study.

## Figures and Tables

**Table 1:** Demographic and clinical features of synchronous COF cases included in the present systematic review.

Variable		n (%)
Continent (n=20)	India	4 (20)
	Turkey	3 (15)
	United States	3 (15)
	Brazil	2 (10)
	China	2 (10)
	Italy	2 (10)
	Japan	2 (10)
	Kenya	1 (5)
	Korea	1 (5)
Sex (n=20)	Female	12 (60)
	Male	8 (40)
Age (years) (n=20)	Mean (SD)	35 (±13.8)
	Range	6 - 55
Anatomical location (n=20)	Maxilla and Mandible	13 (65)
	Mandible	5 (25)
	Maxilla	2 (10)
Treatment (n=17)	Surgical enucleation	7 (41.2)
	Surgical excision	4 (23.5)
	Resection	4 (23.5)
	Symptomatic therapy	1 (5.9)
	Treatment was delayed	1 (5.9)
Recurrence (n=13)	Yes	3 (23.1)
	No	10 (76.9)
Follow-up (months) (n=10)	Mean (SD)	44.7 (±62.19)
	Range	3 - 216

SD, standard deviation.
